# Innate Immune Recognition of *Mycobacterium tuberculosis*

**DOI:** 10.1155/2011/405310

**Published:** 2011-04-07

**Authors:** Johanneke Kleinnijenhuis, Marije Oosting, Leo A. B. Joosten, Mihai G. Netea, Reinout Van Crevel

**Affiliations:** Department of Medicine, Radboud University Nijmegen Medical Centre, and Nijmegen Institute for Infection, Inflammation and Immunity (N4i), Geert Grooteplein Zuid 8, 6525 GA Nijmegen, The Netherlands

## Abstract

Tuberculosis (TB), caused by *Mycobacterium tuberculosis* (MTB), is a major health problem, with 10 million new cases diagnosed each year. Innate immunity plays an important role in the host defense against *M. tuberculosis*, and the first step in this process is recognition of MTB by cells of the innate immune system. Several classes of pattern recognition receptors (PPRs) are involved in the recognition of *M. tuberculosis*, including Toll-like receptors (TLRs), C-type lectin receptors (CLRs), and Nod-like receptors (NLRs). Among the TLR family, TLR2, TLR4, and TLR9 and their adaptor molecule MyD88 play the most prominent roles in the initiation of the immune response against tuberculosis. In addition to TLRs, other PRRs such as NOD2, Dectin-1, Mannose receptor, and DC-SIGN are also involved in the recognition of *M. tuberculosis*. Human epidemiological studies revealed that genetic variation in genes encoding for PRRs and downstream signaling products influence disease susceptibility, severity, and outcome. More insight into PRRs and the recognition of mycobacteria, combined with immunogenetic studies in TB patients, does not only lead to a better understanding of the pathogenesis of tuberculosis but also may contribute to the design of novel immunotherapeutic strategies.

## 1. Introduction

Tuberculosis (TB) is a major public health problem, with 10 million new cases diagnosed each year, causing a death toll of 2 million victims. However, from the estimated 2 billion persons individuals that have been initially infected with *Mycobacterium tuberculosis*, only 5% to 10% develop symptomatic TB. 

The reason why some infected individuals develop active disease while others do not is not yet entirely understood. The role of inborn variability in susceptibility to tuberculosis has been accidentally proven by an episode that occurred almost a century ago, when in 1926 newborn infants from the town of Lübeck in Germany received live *Mycobacterium tuberculosis* (MTB) instead of the vaccine bacillus Calmette-Guérin (BCG). Some of the children became gravely ill, while others were unaffected [1]. This finding indicates that at least some individuals display an effective immune response to MTB and that this plays an important part in determining the outcome of the infection. In addition, this episode in young infants known to have immature adaptive immunity also suggests that the innate host defense is an important arm of antimycobacterial host defense. 

Much has been learned during the last decade on the mechanisms through which the immune response to MTB is initiated. The first step is the recognition of mycobacteria as invading pathogens, followed by activation of innate host defense responses, and the subsequent initiation of adaptive immune responses. Knowledge about these processes is crucial for understanding the pathophysiology of tuberculosis, on the one hand, and for the development of novel strategies of vaccination and treatment such as immunotherapy on the other hand. This paper focuses on the first step of the immune response, which is the recognition of mycobacteria by cells of the innate immune system.

Initiation of the innate immune response starts with pattern recognition of microbial structures called pathogen-associated molecular patterns (PAMPs). Recognition of PAMPs is performed by germline-encoded receptors expressed mainly on immune cells termed pattern recognition receptors (PRRs) [2]. The first step in understanding the mechanisms of recognition of pathogenic bacteria is a solid knowledge of the structure of the cell wall of the microorganism, which is the first structure to come in contact and to be recognized by the cells of the immune system.

### 1.1. The Mycobacterial Cell Wall

**Figure 1:**
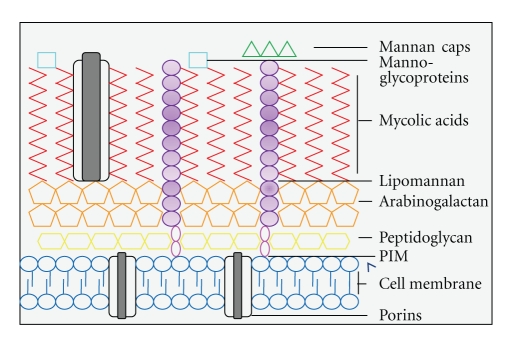
The structure of the *Mycobacterium tuberculosis *cell wall. This figure shows a schematic representation of the major components of the cell wall and their distributions. The inner layer is composted of peptidoglycan which is covalently linked to arabinogalactan layer. The outer membrane contains mycolic acids, glycolipids like (mannose-capped) lipomannan, and mannoglycoproteins.

MTB is a slow-growing intracellular pathogen that can survive inside the macrophage of the host. MTB is an acid-fast bacterium due to the fact that the cell wall mainly consists of hydrophobic mycolic acids. This is a specific component of mycobacterial cell wall and makes up 50% of its dry weight. Due to this thick layer of mycolic acids, the entry of nutrients is impaired, which causes slow growth of mycobacteria, but it also increases cellular resistance to degradation through lysosomal enzymes [3]. The mycolic acids are distributed as a thick layer mostly at the external portions of the cell wall, while the internal layers of mycobacteria consist mostly of arabinogalactan, phosphalidyl-*myo*-inositol mannosides (PIMs), and peptidoglycans (Figure 1) [4]. Next to the mycolic acid layer, other components include mannose-containing biomolecules including mannose-capped lipoarabinomannan (Man-LAM), the related lipomannan (LM), and mannoglycoproteins [4]. Mannan and arabinomannan are present on the surface and form the outer capsule of this bacterium. Man-LAM, LM, and PIMs all share a conserved mannosyl-phosphatidyl-*myo*-inositol (MPI) domain that presumably anchors the structures into the plasma membrane [5]. 

Man-LAM, one of the most abundant mannans present on the cell surface, is an important virulence factor of MTB [6]. Man-LAM is a heterogeneous lipoglycan with a characteristic tripartite structure of a carbohydrate core, the MPI anchor, and various mannose-capping motifs. These mannose-capped motifs are characteristic for all pathogenic mycobacteria, and they are not present on fast-growing mycobacterial strains which are significantly less pathogenic. These strains have either uncapped LM or have phospho-*myo*-inositol caps (PILAM), which are known to display more robust immunostimulatory effects. PIMs can be divided into two groups dependent on the mannose content, which determines its immunogenic effect [7, 8]. Also present on the cell surface are the mannoglycoproteins, which can also be secreted during growth.

### 1.2. Innate Immunity and Host Defense

After the inhalation of infected aerosols into the lungs of the host, the first encounter of mycobacteria is with alveolar resident macrophages. Mycobacteria that escape the initial intracellular destruction can multiply and disrupt the macrophage, after which chemokines are released, attracting monocytes and other inflammatory cells to the lung. Inflammatory monocytes will differentiate into macrophages, which readily ingest but do not destroy the mycobacteria [9]. In this stage of the infection, the mycobacteria grow logarithmically and blood-derived macrophages accumulate, but little tissue damage occurs. Two-to-three weeks after infection, T-cell immunity develops and antigen-specific T lymphocytes arrive, proliferate within the early lesions or tubercles, and release proinflammatory cytokines such as interferon-*γ* (IFN*γ*) that will activate macrophages to kill the intracellular mycobacteria. Subsequently, the early logarithmic bacillary growth stops, and central solid necrosis in these primary lesions or granuloma inhibits extracellular growth of mycobacteria. Several scenarios may follow, with infection becoming stationary or dormant in some individuals, or progressive in the lung, or with hematogenous dissemination in a minority of patients. In addition, reactivation can occur months or years afterwards, under conditions of failing immune surveillance [9]. Granuloma often contains central caseous necrotic tissue, which gives rise to cavities and aerogenic spread of mycobacteria.

The macrophage is a pivotal cell in these events, as it is involved in phagocytosis and killing of mycobacteria as well as in the initiation of adaptive T-cell immunity. Phagocytosis of MTB involves different receptors such as the scavenger receptors, the mannose receptor (MR), and complement receptors [10–13]. Phagocytosis can involve both uptake of the bacilli after opsonization with complement factors, or it can be initiated as a nonopsonic event. *In vitro* experiments have shown that complement receptor 3 (CR3) mediates approximately 80% of complement-opsonized MTB phagocytosis [12]. Nonopsonic phagocytosis is an important process in the primary infection of the lung, because complement factors are largely absent in the alveolar space [14]. 

Macrophages can eliminate mycobacteria through different mechanisms, such as production of reactive oxygen and nitrogen species, acidification of the phagosome, and phagosome fusion with the lysosomes [9]. The fate of intracellular mycobacteria is also influenced by autophagy, a cellular process though which cytoplasmic components, including organelles and intracellular pathogens, are sequestered in a double-membrane-bound autophagosome and delivered to the lysosome for degradation [15]. Activation of autophagy leads to phagosome maturation, an increased acidification in the phagosome, and killing of mycobacteria in macrophages [16]. However, once inside the cell, MTB often evades destruction by the innate microbial machinery [17], one of the main mechanisms being the inhibition of phagosome-lysosome fusion [18]. 

The interaction between MTB and cells of both the innate and adaptive immune system results in the secretion of chemokines and cytokines, the most important being tumor necrosis factor-*α* (TNF*α*), cytokines of the interleukin-1 family (IL-1*β*, IL-18), IL-12, and IFN*γ*. TNF*α*-deficient mice succumb rapidly after MTB infection, with significantly higher mycobacterial outgrowth in different organs compared to wild-type animals [19]. TNF*α* is also important for formation of granuloma, an important mechanism for containing and restricting the replication of the bacilli [19, 20]. The importance of IL-1*β* production is underlined by the fact that intact IL-1-mediated signals are essential components of the host defense to mycobacteria [21–23]. Infection of IL-1 receptor type 1 knockout mice with MTB is associated with lower production of IFN*γ*, defective granuloma formation, and lower survival [22].

IFN*γ* activates macrophages to kill and eliminate the mycobacteria. It also enhances their expression of MHC class II molecules, which results in improved antigen presentation to T cells. IFN*γ* is secreted by NK, CD4+, and CD8+ T cells upon release of endogenous IL-12 and IL-18 by macrophages and dendritic cells. The crucial importance of IFN*γ* for human antimycobacterial defence is demonstrated by the increased susceptibility to mycobacterial infections in patients with IFN*γ* receptor or IL-12 receptor deficiencies [24–26]. 

Various macrophages subsets have been identified with different potential functions. For example, alveolar macrophages, usually the first encounter with the mycobacterium, have an immune suppressive and poor antigen presenting ability [27, 28]. Two main subtypes are described, the classical and the nonclassical or alternative phenotypes. The classical route of differentiation induced by microbial products or IFN*γ* leads to induction of antimicrobial effects and production of proinflammatory cytokines as TNF*α*, IL-1*β*, IL12 (p40), and IL23 [29, 30]. This is in contrast to the nonclassical macrophages subsets, which lack antimicrobial activity and production of IL-12. These subsets have a poor antigen presenting capacity and can suppress cellular immunity by production of IL-10 [30]. The macrophages subset polarization may determine the outcome of the host response in skewing the pro- and anti-inflammatory immune response and subsequently in elimination of mycobacteria.

The first step in the activation of innate host defense begins with the pattern recognition of the pathogen. The PAMPs of MTB are sensed by specific PRRs, which in turn trigger production of proinflammatory cytokines and chemokines, phagocytosis and killing of the mycobacteria, and antigen presentation. This paper focuses on the role of the PRRs and downstream signaling for the recognition of MTB, including the intracellular mechanisms activated by PRRs. First, we will review specific evidence from in vitro studies and animal research. Then, we will discuss the human genetic studies done to assess the role of variation in PRR genes for the susceptibility to tuberculosis.

## 2. Recognition of *Mycobacterium tuberculosis*—Experimental Studies

**Figure 2:**
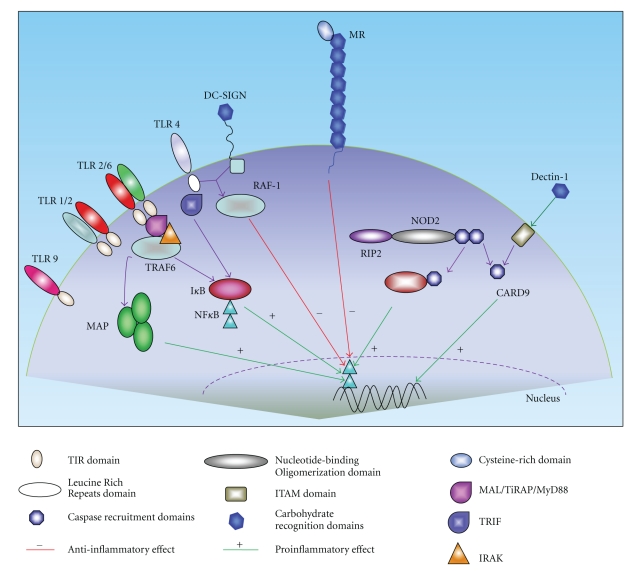
Pattern recognition receptors in the recognition of mycobacteria and downstream signaling pathways. Mycobacteria can be recognized through different pattern recognition receptors (PRRs) of the host. Both intracellular and extracellular receptors are involved in this process. After recognition of mycobacteria, intracellular signaling cascades are activated which eventually will lead to the activation of transcription of NF-*κ*B. After transcription, the production of pro- and anti-inflammatory cytokines and chemokines is induced. The type of signaling cascade induced depends mainly on the type of PRR that recognizes (components of) *MTB*.

The interaction between MTB and host cells is complex and, although extensively studied, not yet completely elucidated. Here we will focus on the PRRs that recognize specific PAMPs of mycobacteria and induce intracellular signals leading to cytokine production and initiation of adaptive immunity. A schematic representation is presented in Figure 2. Host receptors which are mainly involved in bacterial phagocytosis rather than immune recognition, such as complement receptors and scavenger receptors, go beyond the scope of this paper.

### 2.1. Toll-Like Receptors

Toll-like receptors (TLRs) are a family of PRRs consisting of 12 members in mammals. TLRs are expressed on the surface of the cell membrane or on the membrane of endocytic vesicles of mainly immune cells including macrophages and dendritic cells (DCs). Although the interaction of MTB with TLRs leads to phagocyte activation, the interaction itself does not lead to immediate ingestion of the mycobacteria. After the interaction of specific mycobacterial structures with TLRs, signaling pathways are triggered in which adaptor molecule myeloid differentiation primary response protein 88 (MyD88) plays an important role [31]. Subsequently, IL-1 receptor-associated kinases (IRAK), TNF receptor-associated factor (TRAF) 6, TGF*β*-activated protein kinase 1 (TAK1), and mitogen-activated protein (MAP) kinase are recruited in a signaling cascade leading to activation and nuclear translocation of transcription factors such as the nuclear transcription factor (NF)-*κ*B [32]. This leads to the transcription of genes involved in the activation of the innate host defense, mainly the production of proinflammatory cytokines as TNF, IL1*β*, and IL-12 and nitric oxide [33]. 

MyD88 plays a central role in the activation of the innate immune response to *M. tuberculosis*; compared to wild-type mice, MyD88 knockout mice are more susceptible to infection [21]. In addition to MyD88, TLR4 can induce intracellular signals through a second pathway, which is mediated by the adaptor molecule Toll/IL-1R (TIR) domain-containing adapter inducing interferon (IFN)-*β* (TRIF). Recently, this MyD88-independent, TRIF-dependent TLR4-signaling cascade was shown to be involved in the LPS-induced autophagy [34]. As the TLR4-induced activation of autophagy plays an important role in the phagosome-lysosome fusion, a process counteracted by MTB [34], it is tempting to speculate that the interaction between TRIF and autophagy is an important component of the innate host defense to mycobacteria.

The TLRs known to be involved in recognition of MTB are TLR2, TLR4, TLR9, and possibly TLR8 [35–40]. TLR2 forms heterodimers with either TLR1 or TLR6. These heterodimers have been implicated in recognition of mycobacterial cell wall glycolipids like LAM, LM, 38-kDa, and 19-kD mycobacterial glycoprotein, and phosphatidylinositol mannoside (PIM), triacylated (TLR2/TLR1), or diacylated (TLR2/TLR6) lipoproteins [39, 41, 42]. TLR2 is believed to be important in the initiation of innate host defense through its stimulatory effects on TNF*α* production in macrophages [31, 38]. In turn, an important role for TLR2 and TLR6 but not TLR4 or TLR9, was found for the stimulation of IL-1*β* production [43]. TLR2 is also important for IL-12 release in macrophages, but not in DCs [44]. TLR2-/- mice show defective granuloma formation, and when infected with high doses of MTB, they have a greatly enhanced susceptibility to infection compared to the WT mice [45, 46]. In addition, TLR2-/- mice display defects in controlling chronic infection with MTB [46].

TLR4 is activated by heat shock protein 60/65 [37, 47], a protein that is secreted by a variety of MTB species. Studies with TLR4 transfected CHO cells and murine macrophages showed the importance of TLR4 in recognition of MTB [36, 39]. Macrophages of TLR4-deficient mice showed less, but not completely abolished, TNF*α* production. *In vivo* murine studies on the role of TLR4 in the recognition of MTB have shown conflicting results, even when the same mouse strain was used. Reiling et al. showed that TLR4-deficient mice, in contrast to TLR2 deficient mice, showed similar susceptibility to MTB infection compared to wild-type animals [45]. In contrast, Abel et al. reported higher mycobacterial outgrowth in lungs, spleen, and liver and a lower survival following infection compared to wild-type animals [48]. More studies are necessary to elucidate the source of these discrepancies and the role of TLR4 for MTB infection.

TLR9 recognizes unmethylated CpG motifs in bacterial DNA. *In vitro* studies showed that MTB-induced IL-12 release in dendritic cells was TLR9-dependent [38, 44]. *In vivo* experiments showed that when mice were infected with a high infectious dose of MTB, animals lacking TLR9 succumb earlier to infection than wild-type animals [38].

TLR8 is able to recognize single-stranded RNA from pathogens such as RNA viruses. Interestingly, Davila et al. demonstrated upregulation of TLR8 protein expression in macrophages after infection with BCG [40]. Until now, this is the only study addressing a potential role of TLR8, but the mechanism through which TLR8 recognizes MTB and signals intracellular remains unknown.

A partially redundant role of TLRs for the host defence against mycobacteria has been suggested, and it has been hypothesized that defects in multiple TLRs are necessary to unveil the role of these receptors for antimycobacterial defense. Indeed, TLR2 and TLR9 double knockout mice display greater defects of IL-12 and IFN-*γ* production in comparison with both single TLR knockout mice, and they succumb earlier to infection even when infected with a low inoculum of MTB [38].

### 2.2. NOD Like Receptors

The NOD like receptors (NLRs) family of proteins highly resembles the family of plant R (resistance) proteins, which have a crucial role in the defence against plant pathogens. The mammalian NLR family consists of more than twenty members with a conserved structure. The core of the molecule is formed by the nucleotide-binding domain, named NACHT (NAIP, CIITA, HET-E, and TP-1 [49]) or NOD (nucleotide oligomerization domain) domain. The C-terminal part consists of a series of leucin-rich repeats, which are thought to recognize the PAMPs of the pathogen and initiate activation of the molecule. The N-terminal portion of the molecule contains an effector domain of CARD (caspase activation and recruitment domain), PYRIN, or BIR (baculovirus inhibitor of apoptosis repeat domain) [50]. CARD-containing NLRs such as NOD1 and NOD2 are thought to form oligomers and then to recruit receptor-interacting protein 2 (RIP2) (or CARD containing kinase—RICK) through CARD-CARD interactions, which leads to the recruitment of NF-*κ*B [51]. 

A major signalling pathway for the activation of the antimycobacterial host defense is represented by the inflammasome, that through activation of caspase-1 leads to processing of procytokines of the IL-1 family into the bioactive IL-1*β* and IL-18. Several PYRIN-domain containing NLRs (NALPs) can form different variants of the inflammasome containing either NLRP1, NLRP3 (cryopyrin), or NLRC4 (Ipaf) [52], as well as the adaptor protein ASC [53, 54]. A fourth type of inflammasome formed by the intracellular protein AIM2 is activated by intracytoplasmic DNA [55]. A recent study has shown that induction of IL-1*β* production by MTB is mediated by TLR2/TLR6 and NOD2 receptors, while caspase-1 is constitutively activated in human primary monocytes [43]. This is in contrast with the study of Master et al. that suggested that MTB inhibits inflammasome activation and IL-1*β* production [56]. However, these studies are not completely comparable as the latter study has used murine macrophage cell lines, in contrast to the human primary cells used by the former study. In addition, if MTB would inhibit IL-1*β* production even in normal hosts, this could not explain the increased susceptibility to infection of IL-1R-deficient mice [22].

NOD2 is an intracellular receptor-mediating stimulation of proinflammatory cytokine production by MTB. NOD2 is a receptor for bacterial peptidoglycans [57], and recently, we demonstrated its role in the recognition of mycobacteria [58, 59]. NOD2-deficient mice showed impaired production of proinflammatory cytokines and nitric oxide when infected with MTB. However, the susceptibility to MTB infection of NOD2-deficient mice is variable [60, 61].

### 2.3. C-Type Lectins

C-type lectins are a family of PRRs involved in the recognition of polysaccharide structures of pathogens. The mannose receptor (MR, CD206) consists of eight linked carbohydrate recognition domains and one cysteine-rich domain. MR is highly expressed on alveolar macrophages [62]. Mycobacterial stimulation through MR leads to production of the anti-inflammatory cytokines IL-4 and IL-13, inhibition of IL-12 production, and failure to activate oxidative responses [63, 64]. Man-LAM and other major components of the MTB cell wall like PIMs are natural mycobacterial ligands for MR. In addition, binding of MTB to MR induces phagocytosis, but phagosome-lysosome fusion is limited [65–67]. 

Differences at the level of mannosylation between MTB strains may also contribute to recognition by C-type lectins. Torrelles and Schlesinger showed that differences in virulence between MTB strains could be related to expression of Man-LAM on the cell wall [4]. Virulent MTB strains with less surface mannosylation do not use MR for phagocytosis but rely primarily for recognition and phagocytosis on CR3 after opsonisation. These strains are virulent because they display more other cell envelope components (like phenolic glycolipids and triacylglycerols) [68, 69]. These cell components regulate the cytokine response and demonstrate rapid intracellular growth and marked tissue damage [70, 71]. On the contrary, heavily mannosylated MTB strains such as the laboratory strain H37Rv use the MR receptor during invasion of the cell and are associated with a higher survival within the macrophage and an anti-inflammatory cytokine response. It is speculated that this type of recognition might lead to a latent stage of infection [4]. This might not be the case for all mycobacterial species; a mutant *Mycobacterium bovis* strain, which entirely lacked surface mannose, showed a comparable cytokine profile as the nonmutant did [72].

### 2.4. DC-SIGN

Dendritic cell-specific intercellular adhesion molecule-3 grabbing nonintegrin (DC-SIGN, CD209) plays an important role in MTB-DC interaction. This receptor is mainly expressed on DCs and serves as both a PRR and an adhesion receptor, due to its functions in DC migration and DC-T-cell interactions [73, 74]. The carbohydrate recognition domain of DC-SIGN recognizes Man-LAM and lipomannans and the amount of Man-LAM determines the binding strength [64]. Recently, it was shown that *α*-glucan (a dominant capsular polysaccharide) is also a ligand for DC-SIGN [75]. After engagement of mycobacoerial structures, DC-SIGN promotes an anti-inflammatory immune response by maturation of infected DCs and induction of IL-10 production [64]. Later, it was shown that DC-SIGN exerts its immunosuppressive effects through induction of acetylation of the NF-*κ*B subunit p65 via Raf-1, but only in the presence of simultaneous TLR stimulation [76].

### 2.5. Dectin-1

Dectin-1 is a receptor with an extracellular carbohydrate recognition domain and an intracellular ITAM domain. This receptor is mainly expressed on macrophages, DCs, neutrophils, and a subset of T-cells. Dectin-1 mainly recognizes *β*-glucans present in fungal pathogens, but it has been suggested to play an important role in MTB recognition as well. The precise PAMP that leads to the recognition through dectin-1 is not known although some species of MTB express *α*-glucan on the cell surface [77] as a ligand for dectin-1. Murine bone marrow-derived macrophages infected with either virulent or avirulent mycobacteria produce TNF-*α* and IL-6 in a dectin-1-independent or dectin-1-dependent manner, respectively [78]. A study with DCs isolated from spleens showed that dectin-1 triggers the production of IL-12 [79]. Several studies have shown synergistic effects between TLR2 and dectin-1 for the recognition of fungal pathogens [80, 81], but this remains to be demonstrated in case of mycobacteria. Finally, a recent report showed that dectin-1, independent of TLR2 recognition, is important for the innate immunity recognition of MTB and for inducing Th1 and Th17 responses [82].

## 3. Recognition of *Mycobacterium tuberculosis*—Human Genetic Studies

In order to have a complete picture of the role of PRRs for the host defense to MTB, the results of *in vitro* and animal studies need to be corroborated with studies in patients. The association of host genetic factors with susceptibility or resistance to TB has been studied extensively with candidate gene approaches and genome-wide association studies. These analyses have revealed several important candidate genes for susceptibility to TB [83, 84]. For the scope of this paper this section is limited to PRRs and their signaling pathways only.  Table 1 shows an overview of investigated SNPs with or without association with TB.

The TLR2 gene is located on chromosome 4q32 and is composed of two noncoding exons and one coding exon [85]. More than 175 SNPs for the human TLR2 have been reported. In a Turkish cohort, an association between Arg753Gln and susceptibility to TB [86] was reported, while this was not confirmed in two Asian cohorts due to the absence of this particular polymorphism in these populations [87, 88]. Arg753Gln seems to be present only in Caucasian populations, with percentages ranging from 0 to 0,49% in East Asian populations [87, 89–91]. In a Tunisian cohort, Arg677Trp showed an association with susceptibility to TB [92], but these results were put in doubt by the discovery of a pseudogene on which this SNP seems to be located [93].

The TLR2 genotype 597CC has been correlated with susceptibility to TB, especially with disseminated forms of the infection (miliairy and meningitis) caused by a particular MTB genotype family (“the Beijing genotype”), in a cohort of patients from Vietnam [107, 108]. A highly polymorphic guanine-thymine repeat, located 100 base pairs upstream of the TLR2 translation start site in intron 2, was correlated with promoter activity and the expression of TLR2 on CD14+ PBMCs (the shorter the repeat, the weaker the promoter activity and the lower the expression of TLR2) for both tuberculosis and nontuberculosis mycobacterial lung infections in a Korean cohort [109, 110]. However, these data could not be reproduced in a Taiwanese population [111]. Another variation in genotype that seems to influence TLR2 expression is −196 to −174 insertion/deletion, with a recent study displaying an association with TB, while another study showed a possible effect on development of systemic symptoms [96, 111]. Many other polymorphisms in human TLR2 are examined for their association with enhanced susceptibility to TB, but this requires further confirmation [96]. 

Since TLR1 and 6 form heterodimers with TLR2, SNPs in these receptors might influence TLR2 signalling as well. One example is Ile602Ser SNP in TLR1, which leads to aberrant TLR1 cell trafficking, no functional TLR1 on the cell surface, and which might influence the mycobacterial recognition [112]. The 602I variant is overexpressed in African-Americans infected with TB [94]. In addition, an association between the TLR6 SNPs Ser249Pro and Thr361Thr and MTB-induced cytokine production has been shown [100].

Immunogenetic studies have reported in two other TLR genes: TLR4 and TLR8. In these genes, the genetic variation associated with susceptibility to TB seems to be less pronounced. 

TLR4 Asp299Gly SNP showed an association with TB in HIV positive Caucasians and Tanzanians, but not in a Gambian population [97–99]. TLR8 has always been linked with recognition of viral PAMPs, but in an immunogenetic study in Indonesia, the TLR8 gene, which is located on the X chromosome, was the only gene showing an association with TB. This finding was confirmed in a second much larger cohort from Russia and supported by functional data, as discussed above [40]. Further studies are needed to confirm these findings.

Besides the PRR receptor polymorphisms, SNPs in the TLR signaling pathways may also influence susceptibility to MTB. Khor et al. proposed that the Ser180Leu SNP in the gene coding for TIR domain-containing adaptor protein (TIRAP) was associated with a higher susceptibility to TB in a cohort from West Africa [101] although the frequency of the mutant allele was very rare. However, this association could not be confirmed in a study involving 9000 individuals from Ghana, Russia and Indonesia [102].

Regarding the other PRRs important for MTB recognition, the 871G and 336A variants located in the promoter region of DC-SIGN were associated with protection against tuberculosis in a South African cohort of patients [103]. This finding was, however, not confirmed in a Tunisian cohort [105], while a later study even showed an association in opposite direction (a protective effect of 336G) [104]. Furthermore, genetic variation of the neck region of DC-SIGN (which supports the carbohydrate recognition domain) failed to show an association with tuberculosis susceptibility [105, 113].

## 4. Conclusions and Future Research

Pattern recognition of MTB is a complex process in which a multitude of receptors recognize specific PAMPs of the microorganism. Recognition by specific receptors is followed by different intracellular signalling pathways, in order to integrate and induce an efficient activation of the innate host defense mechanisms. While activation through TLRs, NLRs and dectin-1 initiates essentially a proinflammatory response, signalling through the C-type lectins DC-SIGN or MR have mainly a modulatory function. The interplay between these pathways lead to finely tuned response of the immune system during the encounter with MTB.

One has to acknowledge that both *in vitro* and *in vivo* studies suffer from specific limitations, which may at least partly explain some discrepancies between experimental and immunogenetic studies in TB patients. *In vitro* studies use various cell types, murine macrophages (bone-marrow derived or alveolar), DCs or PBMCs. This can influence the outcome due to the preferential expression of specific receptors on different cell types. A second limitation is that in most in vitro studies only a single receptor is examined, isolated from its physiological environment, while the interplay between different pathways is probably one of the most relevant aspects of pathogen recognition. The role of the innate immune receptors involved in MTB recognition has often been studied in transfected cell lines, while animal models deficient of specific receptors show that these receptors can compensate for each other and sometimes display redundant roles [114–116].

*In vivo* animal studies have the disadvantage that the most commonly used murine models do not represent human TB; granuloma are not formed in these models, which is a crucial step in the latency of this disease. Rabbit and monkey models which are more similar with human TB are rarely used. Even human genetic studies have limitations in terms that these studies often lack the translation at the level of protein function, while in other situations, an important gene is highly conserved and lacks functionally relevant genetic variants that can be assessed. 

While pattern recognition is an important component of the host response to infection with MTB, other factors are relevant as well, including the intrinsic capacity of macrophages to kill MTB, the distribution and function of different T-cell subsets, and regenerative and fibrotic tissue responses. These particular aspects were beyond the scope of this paper.

Humans and MTB have coevolved for millennia, and it is likely that a close relationship exist at the genomic level. Indeed, two studies have shown a direct association between the genetic characteristics of patients with tuberculosis and their mycobacterial isolates [108, 117]. Polymorphisms in either TLR2 and SLC11A1 (NRAMP1) were associated with higher change of being infected with strains belonging to the evolutionary successful *M. tuberculosis* Beijing genotype. Globally, *M. tuberculosis* shows strong geographical differences [118, 119], and this might be triggered by evolutionary pressure from the innate immune system (“coevolution”). Besides *M. tuberculosis*, also, host immune gene polymorphisms show strong geographical differences. The studies of Caws et al. and Van Crevel et al. [108, 117] provide support for the hypothesis that evolutionary adaptation of particular *M. tuberculosis* lineages to certain human populations. For instance, in the case of TLR2 in the study of Caws et al., a certain *M. tuberculosis *genotype family might have a higher or lower affinity for TLR2 expressed in individuals with a particular TLR2 genotype, leading to differences in downstream signalling and subsequent events after recognition of *M. tuberculosis*. Clearly, this concept needs to be investigated in terms of innate immune recognition by examining a number of PRR genes in TB patients in relation to their infective *M. tuberculosis *genotypes. Comparing host-mycobacterial genotype relationships of more successful *M. tuberculosis *genotypes like the Beijing family and less successful genotypes will help increase the understanding of the concept of “coevolution”, virulence and innate host defense to *M. tuberculosis.*

Other important new areas of research related to innate immunity have been initiated recently, and their relationship with tuberculosis remains to be answered. One of the important cellular responses associated with antimycobacterial defense has been suggested to be the process of autophagy. Autophagy has been also shown to modulate the inflammation [120], especially through its interaction with the peptidoglycan receptor NOD2 [121, 122]. One important question to be answered is whether there is a role for autophagy in the induction of an inflammatory response by MTB. What is the explanation for the apparent redundancy in the pattern recognition, and which PRR is most important in which stage of the disease? Answers to these questions are needed in order to develop rational immunotherapeutic interventions like addition of TLR-agonists to candidate vaccines.

More can also be learned from studies in human patients. For instance, patients with advanced HIV-infection have virtually no T-cell immunity. However, even in settings which are hyperendemic for TB, some HIV-infected patients will never develop TB. Certainly, these individuals must have a very effective innate host response against MTB. A pivotal approach will be to combine genetic with functional studies; what does a SNP associated with susceptibility to TB mean in terms of the function of the immune response?

Another suggestion is to study an increased number of SNPs in more PRRs in the same population and to assess the cumulative effects of various combinations of SNPs to obtain a stronger association with disease. A striking observation is that only loss-of-function mutations are investigated. Could it be that gain-of-function mutations of PRRs might influence the immune response to MTB as well?

Finally, one of the most important challenges for the coming years is to translate the knowledge gained in the basic science of immune responses to mycobacteria into improved or novel immune-based treatment strategies, ranging from a better vaccine to immunotherapy.

## Figures and Tables

**Table 1:** SNPs associated with susceptibility to tuberculosis.

Receptor/signalling pathway	Gene	Amino acid	Association	No association
TLR1	1805T>G	Lle602Ser	[94]	[95]

TLR2	816T>C	Asn199Asn		
	2258G>A	Arg753Gln	[86]	[95]
	196 to 174 I/D*	—	[96]	[87, 89, 91]

TLR4	13843A>G	Asp299Gly	[97, 98]	[95, 99]
	1196C>T	Thr399Ile		[95, 97]

TLR6	1083G>C	Thr361Thr	[100]	
	745C>T	Ser249Pro	[100]	[95]

TLR8	4959C>G	—	[40]	
	2921A>G	—	[40]	
	3943A>G	—	[40]	
	5088A>G	Met1Val	[40]	

TLR9	588A>G	Lys196Lys	[96]	
	411C>T	His137His	[96]	

TIRAP	539C>T	Ser180Leu	[101]	[102]

DC-SIGN	336A>G	—	[103, 104]	[105, 106]
	601C>T	—		[103, 105]
	871G>C	—	[103]	[105]
	939C>T	—		[103, 105]
	Neck	—	
	region		[103, 105, 106]
	length	

*I/D insertion/deletion.

## References

[1] Dubos RJ (1952). The white plague: tuberculosis, man and society. *The White Plague: Tuberculosis, Man and Society*.

[2] Akira S, Takeda K, Kaisho T (2001). Toll-like receptors: critical proteins linking innate and acquired immunity. *Nature Immunology*.

[3] Brennan PJ, Nikaido H (1995). The envelope of mycobacteria. *Annual Review of Biochemistry*.

[4] Torrelles JB, Schlesinger LS (2010). Diversity in Mycobacterium tuberculosis mannosylated cell wall determinants impacts adaptation to the host. *Tuberculosis*.

[5] Briken V, Porcelli SA, Besra GS, Kremer L (2004). Mycobacterial lipoarabinomannan and related lipoglycans: from biogenesis to modulation of the immune response. *Molecular Microbiology*.

[6] Strohmeier GR, Fenton MJ (1999). Roles of lipoarabinomannan in the pathogenesis of tuberculosis. *Microbes and Infection*.

[7] Villeneuve C, Gilleron M, Maridonneau-Parini I, Daffé M, Astarie-Dequeker C, Etienne G (2005). Mycobacteria use their surface-exposed glycolipids to infect human macrophages through a receptor-dependent process. *Journal of Lipid Research*.

[8] Torrelles JB, DesJardin LE, MacNeil J (2009). Inactivation of Mycobacterium tuberculosis mannosyltransferase pimB reduces the cell wall lipoarabinomannan and lipomannan content and increases the rate of bacterial-induced human macrophage cell death. *Glycobiology*.

[9] Van Crevel R, Ottenhoff THM, Van der Meer JWM (2002). Innate immunity to Mycobacterium tuberculosis. *Clinical Microbiology Reviews*.

[10] Weikert LF, Edwards K, Chroneos ZC, Hager C, Hoffman L, Shepherd VL (1997). SP-A enhances uptake of bacillus Calmette-Guerin by macrophages through a specific SP-A receptor. *American Journal of Physiology*.

[11] Hirsch CS, Ellner JJ, Russell DG, Rich EA (1994). Complement receptor-mediated uptake and tumor necrosis factor-*α*-mediated growth inhibition of Mycobacterium tuberculosis by human alveolar macrophages. *Journal of Immunology*.

[12] Schlesinger LS, Bellinger-Kawahara CG, Payne NR, Horwitz MA (1990). Phagocytosis of Mycobacterium tuberculosis is mediated by human monocyte complement receptors and complement component C3. *Journal of Immunology*.

[13] Roecklein JA, Swartz RP, Yeager H (1992). Nonopsonic uptake of Mycobacterium avium complex by human monocytes and alveolar macrophages. *Journal of Laboratory and Clinical Medicine*.

[14] Schluger NW (2001). Recent advances in our understanding of human host responses to tuberculosis. *Respiratory Research*.

[15] Kundu M, Thompson CB (2008). Autophagy: basic principles and relevance to disease. *Annual Review of Pathology*.

[16] Gutierrez MG, Master SS, Singh SB, Taylor GA, Colombo MI, Deretic V (2004). Autophagy is a defense mechanism inhibiting BCG and Mycobacterium tuberculosis survival in infected macrophages. *Cell*.

[17] Armstrong JA, D’Arcy Hart P (1975). Phagosome lysosome interactions in cultured macrophages infected with virulent tubercle bacilli. Reversal of the usual nonfusion pattern and observations on bacterial survival. *Journal of Experimental Medicine*.

[18] Armstrong JA, Hart PD (1971). Response of cultured macrophages to Mycobacterium tuberculosis, with observations on fusion of lysosomes with phagosomes. *The Journal of Experimental Medicine*.

[19] Flynn JL, Goldstein MM, Chan J (1995). Tumor necrosis factor-*α* is required in the protective immune response against mycobacterium tuberculosis in mice. *Immunity*.

[20] Kindler V, Sappino AP, Grau GE, Piguet PF, Vassalli P (1989). The inducing role of tumor necrosis factor in the development of bactericidal granulomas during BCG infection. *Cell*.

[21] Fremond CM, Togbe D, Doz E (2007). IL-1 receptor-mediated signal is an essential component of MyD88-dependent innate response to Mycobacterium tuberculosis infection. *Journal of Immunology*.

[22] Juffermans NP, Florquin S, Camoglio L (2000). Interleukin-1 signaling is essential for host defense during murine pulmonary tuberculosis. *Journal of Infectious Diseases*.

[23] Yamada H, Mizumo S, Horai R, Iwakura Y, Sugawara I (2000). Protective role of interleukin-1 in mycobacterial infection in IL-1 *α*/*β* double-knockout mice. *Laboratory Investigation*.

[24] Altare F, Durandy A, Lammas D (1998). Impairment of mycobacterial immunity in human interleukin-12 receptor deficiency. *Science*.

[25] De Jong R, Altare F, Haagen IA (1998). Severe mycobacterial and Salmonella infections in interleukin-12 receptor-deficient patients. *Science*.

[26] Ottenhoff THM, Verreck FAW, Lichtenauer-Kaligis EGR, Hoeve MA, Sanal O, Van Dissel JT (2002). Genetics, cytokines and human infectious disease: lessons from weakly pathogenic mycobacteria and salmonellae. *Nature Genetics*.

[27] Lyons CR, Ball EJ, Toews GB, Weissler JC, Stastny P, Lipscomb MF (1986). Inability of human alveolar macrophages to stimulate resting T cells correlates with decreased antigen-specific T cell-macrophage binding. *Journal of Immunology*.

[28] Blumenthal RL, Campbell DE, Hwang P, DeKruyff RH, Frankel LR, Umetsu DT (2001). Human alveolar macrophages induce functional inactivation in antigen-specific CD4 T cells. *Journal of Allergy and Clinical Immunology*.

[29] Verreck FAW, De Boer T, Langenberg DML (2004). Human IL-23-producing type 1 macrophages promote but IL-10-producing type 2 macrophages subvert immunity to (myco)bacteria. *Proceedings of the National Academy of Sciences of the United States of America*.

[30] Verreck FAW, De Boer T, Langenberg DML, Van Der Zanden L, Ottenhoff THM (2006). Phenotypic and functional profiling of human proinflammatory type-1 and anti-inflammatory type-2 macrophages in response to microbial antigens and IFN-*γ*- and CD40L-mediated costimulation. *Journal of Leukocyte Biology*.

[31] Underhill DM, Ozinsky A, Smith KD, Aderem A (1999). Toll-like receptor-2 mediates mycobacteria-induced proinflammatory signaling in macrophages. *Proceedings of the National Academy of Sciences of the United States of America*.

[32] Takeda K, Akira S (2004). TLR signaling pathways. *Seminars in Immunology*.

[33] Akira S (2003). Toll-like receptor signaling. *Journal of Biological Chemistry*.

[34] Xu Y, Jagannath C, Liu XD, Sharafkhaneh A, Kolodziejska KE, Eissa NT (2007). Toll-like receptor 4 is a sensor for autophagy associated with innate immunity. *Immunity*.

[35] Tapping RI, Tobias PS (2003). Mycobacterial lipoarabinomannan mediates physical interactions between TLR1 and TLR2 to induce signaling. *Journal of Endotoxin Research*.

[36] Means TK, Wang S, Lien E, Yoshimura A, Golenbock DT, Fenton MJ (1999). Human Toll-like receptors mediate cellular activation by Mycobacterium tuberculosis. *Journal of Immunology*.

[37] Bulut Y, Michelsen KS, Hayrapetian L (2005). Mycobacterium tuberculosis heat shock proteins use diverse toll-like receptor pathways to activate pro-inflammatory signals. *Journal of Biological Chemistry*.

[38] Bafica A, Scanga CA, Feng CG, Leifer C, Cheever A, Sher A (2005). TLR9 regulates Th1 responses and cooperates with TLR2 in mediating optimal resistance to Mycobacterium tuberculosis. *Journal of Experimental Medicine*.

[39] Means TK, Jones BW, Schromm AB (2001). Differential effects of a Toll-like receptor antagonist on Mycobacterium tuberculosis-induced macrophage responses. *Journal of Immunology*.

[40] Davila S, Hibberd ML, Dass RH (2008). Genetic association and expression studies indicate a role of Toll-like receptor 8 in pulmonary tuberculosis. *PLoS Genetics*.

[41] Jones BW, Means TK, Heldwein KA (2001). Different Toll-like receptor agonists induce distinct macrophage responses. *Journal of Leukocyte Biology*.

[42] Thoma-Uszynski S, Stenger S, Takeuchi O (2001). Induction of direct antimicrobial activity through mammalian toll-like receptors. *Science*.

[43] Kleinnijenhuis J, Joosten LAB, van de Veerdonk FL (2009). Transcriptional and inflammasome-mediated pathways for the induction of IL-1*β* production by Mycobacterium tuberculosis. *European Journal of Immunology*.

[44] Pompei L, Jang S, Zamlynny B (2007). Disparity in IL-12 release in dendritic cells and macrophages in response to Mycobacterium tuberculosis is due to use of distinct TLRs. *Journal of Immunology*.

[45] Reiling N, Hölscher C, Fehrenbach A (2002). Cutting edge: toll-like receptor (TLR)2- and TLR4-mediated pathogen recognition in resistance to airborne infection with Mycobacterium tuberculosis. *Journal of Immunology*.

[46] Drennan MB, Nicolle D, Quesniaux VJF (2004). Toll-like receptor 2-deficient mice succumb to Mycobacterium tuberculosis infection. *American Journal of Pathology*.

[47] Ohashi K, Burkart V, Flohé S, Kolb H (2000). Cutting edge: heat shock protein 60 is a putative endogenous ligand of the toll-like receptor-4 complex. *Journal of Immunology*.

[48] Abel B, Thieblemont N, Quesniaux VJF (2002). Toll-like receptor 4 expression is required to control chronic Mycobacterium tuberculosis infection in mice. *Journal of Immunology*.

[49] Shi S, Nathan C, Schnappinger D (2003). MyD88 primes macrophages for full-scale activation by interferon-*γ* yet mediates few responses to Mycobacterium tuberculosis. *Journal of Experimental Medicine*.

[50] Proell M, Riedl SJ, Fritz JH, Rojas AM, Schwarzenbacher R (2008). The Nod-Like Receptor (NLR) family: a tale of similarities and differences. *PLoS One*.

[51] Dufner A, Pownall S, Mak TW (2006). Caspase recruitment domain protein 6 is a microtubule-interacting protein that positively modulates NF-*κ*B activation. *Proceedings of the National Academy of Sciences of the United States of America*.

[52] Martinon F, Tschopp J (2004). Inflammatory caspases: linking an intracellular innate immune system to autoinflammatory diseases. *Cell*.

[53] Martinon F, Agostini L, Meylan E, Tschopp J (2004). Identification of bacterial muramyl dipeptide as activator of the NALP3/Cryopyrin inflammasome. *Current Biology*.

[54] Martinon F (2007). Orchestration of pathogen recognition by inflammasome diversity: variations on a common theme. *European Journal of Immunology*.

[55] Burckstummer T, Baumann C, Blüml S (2009). An orthogonal proteomic-genomic screen identifies AIM2 as a cytoplasmic DNA sensor for the inflammasome. *Nature Immunology*.

[56] Master SS, Rampini SK, Davis AS (2008). Mycobacterium tuberculosis prevents inflammasome activation. *Cell Host and Microbe*.

[57] Girardin SE, Boneca IG, Viala J (2003). Nod2 is a general sensor of peptidoglycan through muramyl dipeptide (MDP) detection. *Journal of Biological Chemistry*.

[58] Ferwerda G, Kullberg BJ, De Jong DJ (2007). Mycobacterium paratuberculosis is recognized by Toll-like receptors and NOD2. *Journal of Leukocyte Biology*.

[59] Ferwerda G, Girardin SE, Kullberg BJ (2005). NOD2 and toll-like receptors are nonredundant recognition systems of Mycobacterium tuberculosis. *PLoS Pathogens*.

[60] Gandotra S, Jang S, Murray PJ, Salgame P, Ehrt S (2007). Nucleotide-binding oligomerization domain protein 2-deficient mice control infection with Mycobacterium tuberculosis. *Infection and Immunity*.

[61] Divangahi M, Mostowy S, Coulombe F (2008). NOD2-deficient mice have impaired resistance to Mycobacterium tuberculosis infection through defective innate and adaptive immunity. *Journal of Immunology*.

[62] Gordon S (2003). Alternative activation of macrophages. *Nature Reviews Immunology*.

[63] Nigou J, Zelle-Rieser C, Gilleron M, Thurnher M, Puzo G (2001). Mannosylated lipoarabinomannans inhibit IL-12 production by human dendritic cells: evidence for a negative signal delivered through the mannose receptor. *Journal of Immunology*.

[64] Geijtenbeek TBH, Van Vliet SJ, Koppel EA (2003). Mycobacteria target DC-SIGN to suppress dendritic cell function. *Journal of Experimental Medicine*.

[65] Vergne I, Chua J, Deretic V (2003). Tuberculosis toxin blocking phagosome maturation inhibits a novel Ca2+/calmodulin-PI3K hVPS34 cascade. *Journal of Experimental Medicine*.

[66] Hmama Z, Sendide K, Talal A, Garcia R, Dobos K, Reiner NE (2004). Quantitative analysis of phagolysosome fusion in intact cells: inhibition by mycobacterial lipoarabinomannan and rescue by an 1*α*,25-dihydroxyvitamin D3-phosphoinositide 3-kinase pathway. *Journal of Cell Science*.

[67] Kang PB, Azad AK, Torrelles JB (2005). The human macrophage mannose receptor directs Mycobacterium tuberculosis lipoarabinomannan-mediated phagosome biogenesis. *Journal of Experimental Medicine*.

[68] Reed MB, Domenech P, Manca C (2004). A glycolipid of hypervirulent tuberculosis strains that inhibits the innate immune response. *Nature*.

[69] Reed MB, Gagneux S, DeRiemer K, Small PM, Barry CE (2007). The W-Beijing lineage of Mycobacterium tuberculosis overproduces triglycerides and has the DosR dormancy regulon constitutively upregulated. *Journal of Bacteriology*.

[70] Manca C, Tsenova L, Bergtold A (2001). Virulence of a Mycobacterium tuberculosis clinical isolate in mice is determined by failure to induce Th1 type immunity and is associated with induction of IFN-*α*/*β*. *Proceedings of the National Academy of Sciences of the United States of America*.

[71] Tsenova L, Ellison E, Harbacheuski R (2005). Virulence of selected Mycobacterium tuberculosis clinical isolates in the rabbit model of meningitis is dependent on phenolic glycolipid produced by the bacilli. *Journal of Infectious Diseases*.

[72] Appelmelk BJ, den Dunnen J, Driessen NN (2008). The mannose cap of mycobacterial lipoarabinomannan does not dominate the Mycobacterium-host interaction. *Cellular Microbiology*.

[73] Geijtenbeek TBH, Torensma R, Van Vliet SJ (2000). Identification of DC-SIGN, a novel dendritic cell-specific ICAM-3 receptor that supports primary immune responses. *Cell*.

[74] Geijtenbeek TBH, Krooshoop DJEB, Bleijs DA (2000). DC-SIGN-1CAM-2 interaction mediates dendritic cell trafficking. *Nature Immunology*.

[75] Geurtsen J, Chedammi S, Mesters J (2009). Identification of mycobacterial *α*-glucan as a novel ligand for DC-SIGN: involvement of mycobacterial capsular polysaccharides in host immune modulation. *Journal of Immunology*.

[76] Gringhuis SI, den Dunnen J, Litjens M, van het Hof B, van Kooyk Y, Geijtenbeek TH (2007). C-type lectin DC-SIGN modulates Toll-like receptor signaling via Raf-1 kinase-dependent acetylation of transcription factor NF-kappaB. *Immunity*.

[77] Dinadayala P, Lemassu A, Granovski P, Cérantola S, Winter N, Daffé M (2004). Revisiting the structure of the anti-neoplastic glucans of Mycobacterium bovis Bacille Calmette-Guérin: structural analysis of the extracellular and boiling water extract-derived glucans of the vaccine substrains. *Journal of Biological Chemistry*.

[78] Yadav M, Schorey JS (2006). The *β*-glucan receptor dectin-1 functions together with TLR2 to mediate macrophage activation by mycobacteria. *Blood*.

[79] Rothfuchs AG, Bafica A, Feng CG (2007). Dectin-1 interaction with Mycobacterium tuberculosis leads to enhanced IL-12p40 production by splenic dendritic cells. *Journal of Immunology*.

[80] Gantner BN, Simmons RM, Canavera SJ, Akira S, Underhill DM (2003). Collaborative induction of inflammatory responses by dectin-1 and toll-like receptor 2. *Journal of Experimental Medicine*.

[81] Brown GD, Herre J, Williams DL, Willment JA, Marshall ASJ, Gordon S (2003). Dectin-1 mediates the biological effects of *β*-glucans. *Journal of Experimental Medicine*.

[82] Van De Veerdonk FL, Teirlinck AC, Kleinnijenhuis J (2010). Mycobacterium tuberculosis induces IL-17A responses through TLR4 and dectin-1 and is critically dependent on endogenous IL-1. *Journal of Leukocyte Biology*.

[83] Hill AVS (2006). Aspects of genetic susceptibility to human infectious diseases. *Annual Review of Genetics*.

[84] Bellamy R (2006). Genome-wide approaches to identifying genetic factors in host susceptibility to tuberculosis. *Microbes and Infection*.

[85] Aderem A, Ulevitch RJ (2000). Toll-like receptors in the induction of the innate immune response. *Nature*.

[86] Ogus AC, Yoldas B, Ozdemir T (2004). The Arg753Gln polymorphism of the human Toll-like receptor 2 gene in tuberculosis disease. *European Respiratory Journal*.

[87] Xue Y, Zhao ZQ, Wang HJ (2010). Toll-like receptors 2 and 4 gene polymorphisms in a southeastern Chinese population with tuberculosis. *International Journal of Immunogenetics*.

[88] Biswas D, Gupta SK, Sindhwani G, Patras A (2009). TLR2 polymorphisms, Arg753Gln and Arg677Trp, are not associated with increased burden of tuberculosis in Indian patients. *BMC Research Notes*.

[89] Ma M-J, Xie L-P, Wu S-C (2010). Toll-like receptors, tumor necrosis factor-*α*, and interleukin-10 gene polymorphisms in risk of pulmonary tuberculosis and disease severity. *Human Immunology*.

[90] Cheng PL, Eng HL, Chou MH, You HL, Lin TM (2007). Genetic polymorphisms of viral infection-associated Toll-like receptors in Chinese population. *Translational Research*.

[91] Yoon HJ, Choi JY, Kim CO (2006). Lack of toll-like receptor 4 and 2 polymorphisms in Korean patients with bacteremia. *Journal of Korean Medical Science*.

[92] Ben-Ali M, Barbouche MR, Bousnina S, Chabbou A, Dellagi K (2004). Toll-like receptor 2 Arg677Trp polymorphism is associated with susceptibility to tuberculosis in Tunisian patients. *Clinical and Diagnostic Laboratory Immunology*.

[93] Malhotra D, Relhan V, Reddy BSN, Bamezai R (2005). TLR2 Arg677Trp polymorphism in leprosy: revisited. *Human Genetics*.

[94] Ma X, Liu Y, Gowen BB, Graviss EA, Clark AG, Musser JM (2007). Full-exon resequencing reveals toll-like receptor variants contribute to human susceptibility to tuberculosis disease. *PLoS One*.

[95] Selvaraj P, Harishankar M, Singh B, Jawahar MS, Banurekha VV (2010). Toll-like receptor and TIRAP gene polymorphisms in pulmonary tuberculosis patients of South India. *Tuberculosis*.

[96] Velez DR, Wejse C, Stryjewski ME (2010). Variants in toll-like receptors 2 and 9 influence susceptibility to pulmonary tuberculosis in Caucasians, African-Americans, and West Africans. *Human Genetics*.

[97] Pulido I, Leal M, Genebat M, Pacheco YM, Sáez ME, Soriano-Sarabia N (2010). The TLR4 ASP299GLY polymorphism is a risk factor for active tuberculosis in Caucasian HIV-infected patients. *Current HIV Research*.

[98] Ferwerda B, Kibiki GS, Netea MG, Dolmans WMV, Van Der Ven AJ (2007). The toll-like receptor 4 Asp299Gly variant and tuberculosis susceptibility in HIV-infected patients in Tanzania. *AIDS*.

[99] Newport MJ, Allen A, Awomoyi AA (2004). The toll-like receptor 4 Asp299Gly variant: no influence on LPS responsiveness or susceptibility to pulmonary tuberculosis in The Gambia. *Tuberculosis*.

[100] Shey MS, Randhawa AK, Bowmaker M (2010). Single nucleotide polymorphisms in toll-like receptor 6 are associated with altered lipopeptide- and mycobacteria-induced interleukin-6 secretion. *Genes and Immunity*.

[101] Khor CC, Chapman SJ, Vannberg FO (2007). A Mal functional variant is associated with protection against invasive pneumococcal disease, bacteremia, malaria and tuberculosis. *Nature Genetics*.

[102] Nejentsev S, Thye T, Szeszko JS (2008). Analysis of association of the TIRAP (MAL) S180L variant and tuberculosis in three populations. *Nature Genetics*.

[103] Barreiro LB, Neyrolles O, Babb CL (2006). Promoter variation in the DC-SIGN-encoding gene CD209 is associated with tuberculosis. *PLoS Medicine*.

[104] Vannberg FO, Chapman SJ, Khor CC (2008). CD209 genetic polymorphism and tuberculosis disease. *PLoS One*.

[105] Ben-Ali M, Barreiro LB, Chabbou A (2007). Promoter and neck region length variation of DC-SIGN is not associated with susceptibility to tuberculosis in Tunisian patients. *Human Immunology*.

[106] Gómez LM, Anaya JM, Sierra-Filardi E, Cadena J, Corbi A, Martin J (2006). Analysis of DC-SIGN (CD209) functional variants in patients with tuberculosis. *Human Immunology*.

[107] Thuong NTT, Hawn TR, Thwaites GE (2007). A polymorphism in human TLR2 is associated with increased susceptibility to tuberculous meningitis. *Genes and Immunity*.

[108] Caws M, Thwaites G, Dunstan S (2008). The influence of host and bacterial genotype on the development of disseminated disease with Mycobacterium tuberculosis. *PLoS Pathogens*.

[109] Yim JJ, Lee HW, Lee HS (2006). The association between microsatellite polymorphisms in intron II of the human Toll-like receptor 2 gene and tuberculosis among Koreans. *Genes and Immunity*.

[110] Yim JJ, Kim HJ, Kwon OJ, Koh WJ (2008). Association between microsatellite polymorphisms in intron II of the human Toll-like receptor 2 gene and nontuberculous mycobacterial lung disease in a Korean population. *Human Immunology*.

[111] Chen YC, Hsiao CC, Chen CJ (2010). Toll-like receptor 2 gene polymorphisms, pulmonary tuberculosis, and natural killer cell counts. *BMC Medical Genetics*.

[112] Johnson CM, Lyle EA, Omueti KO (2007). Cutting edge: a common polymorphism impairs cell surface trafficking and functional responses of TLR1 but protects against leprosy. *Journal of Immunology*.

[113] Barreiro LB, Neyrolles O, Babb CL (2007). Length variation of DC-SIGN and L-SIGN neck-region has no impact on tuberculosis susceptibility. *Human Immunology*.

[114] Zimmerli S, Edwards S, Ernst JD (1996). Selective receptor blockade during phagocytosis does not alter the survival and growth of Mycobacterium tuberculosis in human macrophages. *American Journal of Respiratory Cell and Molecular Biology*.

[115] Ehlers S, Reiling N, Gangloff S, Woltmann A, Goyert S (2001). Mycobacterium avium infection in CD14-deficient mice fails to substantiate a significant role for CD14 in antimycobacterial protection or granulomatous inflammation. *Immunology*.

[116] Hu C, Mayadas-Norton T, Tanaka K, Chan J, Salgame P (2000). Mycobacterium tuberculosis infection in complement receptor 3-deficient mice. *Journal of Immunology*.

[117] Van Crevel R, Parwati I, Sahiratmadja E (2009). Infection with mycobacterium tuberculosis Beijing genotype strains is associated with polymorphisms in SLC11A1/NRAMP1 in Indonesian patients with tuberculosis. *Journal of Infectious Diseases*.

[118] Van Soolingen D, Qian L, De Haas PEW (1995). Predominance of a single genotype of Mycobacterium tuberculosis in countries of East Asia. *Journal of Clinical Microbiology*.

[119] Parwati I, Van Crevel R, Sudiro M (2008). Mycobacterium tuberculosis population structures differ significantly on two Indonesian islands. *Journal of Clinical Microbiology*.

[120] Saitoh T, Fujita N, Jang MH (2008). Loss of the autophagy protein Atg16L1 enhances endotoxin-induced IL-1*β* production. *Nature*.

[121] Cooney R, Baker J, Brain O (2010). NOD2 stimulation induces autophagy in dendritic cells influencing bacterial handling and antigen presentation. *Nature Medicine*.

[122] Travassos LH, Carneiro LAM, Ramjeet M (2010). Nod1 and Nod2 direct autophagy by recruiting ATG16L1 to the plasma membrane at the site of bacterial entry. *Nature Immunology*.

